# Supplementary material for: How do medical students deal with the topic of racism? A qualitative analysis of group discussions in Germany
Source: PLoS One. 2024 Nov 19;19(11):e0313614. doi: 10.1371/journal.pone.0313614 (PMC11575774; doi:10.1371/journal.pone.0313614)
Supplement: S1 Text — (DOCX) [file pone.0313614.s001.docx]

**S1: Discussion guide for semi-structured online group discussions**

How do medical students deal with the topic of racism?
A qualitative analysis of group discussions in Germany

Simon Matteo Gerhards, Mark Schweda

1. Introduction:
   1. Please introduce yourself shortly in one or two sentences. What is your background and what motivated you to participate in this study?
2. General perceptions of discrimination in medicine
   1. In your opinion, how far are certain groups disadvantaged in medicine?
3. Personal experiences with racism
   1. Now we would like to focus more in-depth on racism in medicine. Firstly, we will look at your personal experiences with this topic. Please think back: have you noticed situations in the context of medicine and healthcare where racism has played a role?
   2. What was racist about these situations?
   3. Where does racism play a role in medicine and healthcare in Germany? Who is affected by racism in a medical context? (Optional questions)
4. Supraindividual levels of racism in medicine

Discussion prompt: Present graphics with data differentiated by race/ethnicity regarding infant mortality rates, as well as death rates in context of heart disease and COVID19 in the USA (see below).

- 1. Please look at these charts and describe what you see. What do you think are possible reasons for these data distributions?
  2. To what extent are there similar phenomena in Germany?

1. Racism in medical education
   1. Finally, we would like to discuss the role of medical education in relation to this topic. To what extent should racism be addressed in medical studies?
   2. How should this be done?
2. Conclusion
   1. Is there anything we have not talked about yet that you find important to mention with regard to this topic?
   2. Is there any other feedback that you would like to give us that might be useful for our study?

Discussion prompt: supraindividual levels of racism

Based on: National Center for Health Statistics (NCHS) (2016) Health, United States, 2015: With Special Feature on Racial and Ethnic Health Disparities. National Center for Health Statistics (US), Hyattsville (MD), p. 86 (Table 10)

Based on: Rossen LM, Branum AM, Ahmad FB, Sutton P, Anderson RN (2020) Excess Deaths Associated with COVID-19, by Age and Race and Ethnicity - United States, January 26-October 3, 2020. MMWR Morbidity and Mortality weekly report 69 (42):1522-1527. doi:10.15585/mmwr.mm6942e2

Based on: National Center for Health Statistics (NCHS) (2016) Health, United States, 2015: With Special Feature on Racial and Ethnic Health Disparities. National Center for Health Statistics (US), Hyattsville (MD), p. 99 (Table 17)
