# Supplementary material for: How do medical students deal with the topic of racism? A qualitative analysis of group discussions in Germany
Source: PLoS One. 2024 Nov 19;19(11):e0313614. doi: 10.1371/journal.pone.0313614 (PMC11575774; doi:10.1371/journal.pone.0313614)
Supplement: Attachment — Submitted filename: Response to Reviewers.pdf [file pone.0313614.s002.pdf]

Dear Dr. Uyheng,

We would like to thank you, once again, for sharing the reviews of our manuscript „Medical student's habitual approaches to discussions about Racism in Germany" (PONE-D-23-43307) and for giving us the opportunity to revise the text accordingly. The detailed comments and very instructive suggestions helped us tremendously to clarify our conceptual framework and to organize the presentation of our empirical results, thus improving the text considerably. We hereby submit the revised version together with a point-by-point response to the reviewers. We hope the piece now meets the requirements of PLOSone and look forward to hearing from you.

Kind regards,

The Authors

Reviewer #1

Reviewer#1 stated that our article offers important contributions to the field of racism and racialization in medicine and represents an important step towards understanding how racialization works in health care. At the same time, the reviewer pointed out that more work is needed, especially with regard to the definition of our central concepts and the structure of the results.

<u>Main comments:</u>

Reviewer #1 pointed out that our conceptual framework was a bit unclear since important concepts were not defined in the introduction and further concepts introduced in other sections of the text. In particular, this concerned the concepts of habitus and habitual approach, racialized categorization, antiracism, medical professionalization (and its connection to habitus). The reviewer suggested that we go through the text and identify a limited number of core concepts for analysis that are defined in the introduction and form our conceptual framework.

➔ *We would like to thank the reviewer for this very helpful comment and the instructive suggestions! In order to clarify the conceptual framework of our analysis, we made three revisions: As suggested by the reviewer, we first reduced the number of concepts to the core conceptual framework, eliminating concepts that were not essential for our analysis, especially "pointed labels" (marked-up version: l. 380) or "heuristic tools" (marked up version: l. 568, 722, 981, 1077). We replaced the terms "habitual approach" and "medical professionalisation" with the more precise terms of "ways of dealing with" and "professional socialisation". Secondly, we now provide short working definitions of all the main concepts in the introduction: antiracism (lines 36-38, now and in the following lines refer to the unmarked version), professional socialisation (l. 43-45) habitus (line 46), orientation of action (l. 47-48), racism (l. 87-91), races (l. 92-94), racialisation (l. 93-95) and racialised (lines 95-98). Thirdly, we elaborate a bit further on these concepts and their interconnections in the methods section (l. 240-250), also providing a concise overview of our conceptual framework for analysis in the form of a table (l. 151).*

Reviewer #1 noted that the aim of the article was not stated in a consistent way, pointing to varying formulations throughout the text. The reviewer pointed out that different formulations of aims require different methods and different ways of organizing and structuring the results and

recommended to stick with the formulation of the aim stated in the abstract ("to analyse how medical students in Germany deal with the topic of racism in discussions").

➔ *We can see how these different formulations of the article's aims can appear confusing and even misleading. We followed the reviewer's recommendation to stick with the formulation in the abstract that we aim to analyse "how medical students in Germany deal with the topic of racism" (lines 4-5, l. 66-67, l. 166). In line with this formulation, we now state consistently throughout the manuscript that the focus is on analysing medical students' ways of dealing with the topic of racism in discussions (Introduction line 74, methods l. 158, 257, 271, 285). To further increase coherence, we also revised the headings in the results section accordingly and renamed the five "habitual approaches" identified in the analysis and presented in the section as five different "ways of dealing with the topic of racism in discussions", namely a "scientistic way" (ll. 298), a "pragmatic way" (ll. 389), a "subjectivist way" (l. 474), an "interculturalist way" (ll. 547), and a "critical way" (ll. 645). In light of the aforementioned changes, we eventually also decided to adapt the manuscript's title accordingly. It now reads "How do medical students deal with the topic of racism? A qualitative analysis of group discussions in Germany" (l.1-2)*

Reviewer #1 criticized that separating the analysis of performative and interactional aspects of medical students' ways of dealing with the topic of racism in discussions was unnecessary and did not add anything to the article since these aspects are integral to focus groups as such and form part of their analysis anyway. The reviewer recommended to remove these separate titles and integrate the respective aspects in the analysis of each type.

➔ *We would like to thank the reviewer for this suggestion that makes the results section much more integrated and readable. We followed the advice and removed the respective subtitles delineating the analysis of performative and interactional aspects in the results section. The description of each way of dealing with the topic of racism in discussions now integrates performative and interactional aspects throughout, for example l. 335-337 and l. 375-376 for type A, l. 462-473 for type B, l. 537-456 for type C, l. l. 595-607 for type D, l. 681-687 for type E.*

Reviewer #1 also pointed out that the definition of the types provided at the beginning of the results section was not always consistent with what the description of the results subsequently actually showed. The reviewer suggested formulating the definition of the types in a way that is coherent with the respective description of results.

➔ *Thank you for pointing out this inconsistency! We have deleted the whole passage at the beginning of the results section that was aimed to give a short summary overview of the different ways medical students deal with the topic of racism in discussions (l. 367-380 in the marked version). Furthermore, we also made efforts to provide short descriptions of each type that capture its main defining aspects as they are subsequently unfolded in the respective part of the results section (see below).*

Overall, reviewer #1 remarked that there was a jump of logic between the first three and the last two types described in the results section since the former referred to different understandings of 'race' and racism whereas the latter rather addressed different ways to approach 'race' and racism.

➔ *We would like to thank the reviewer very much for addressing this crucial point. We now make clearer right from the start that our analysis and the identified ways of dealing with the topic of racism actually encompass both aspects: different understandings of 'race' and racism as well as different attitudes towards 'race' and racism that both can be related to an underlying*

orientation of action. Thus, we now explain in more detail in the introduction (l. 42-48, l. 133) and the methods section (l. 240-250, 269-289) that our analysis of medical students' habitual ways of dealing with the topic of racism in discussions was aimed at both explicit understandings as well as more or less implicit orientations. We also revised the descriptions of each type to make sure that each description actually explicitly addresses these different aspects. For example, we now make clearer that the 'scientistic' (type A, ll. 298), 'pragmatic' (type B, ll. 379) as well as 'subjectivistic' (type C, ll. 474) (formerly 'relativistic') way are not only about understandings of 'race' and racism, but also about more implicit orientations. Likewise, we point out that the 'interculturalist' (type D, ll. 547) and 'critical' (type E, ll. 645) way are not merely about attitudes or approaches towards 'race' and racism, but also include specific understandings (see below). Finally, this is also mirrored in our rephrased definitions of each type which now include all these aspects (see below).

In particular, reviewer #1 recommended to define 'scientism' in a way that makes clear that this type views 'race' as an objective biological reality and a scientific concept.

➔ *We have changed the definition of 'scientism' accordingly to include this aspect. The respective definition now explains that a scientistic way of dealing with the topic of racism "orientates action towards the idea of medicine as an objective science, justifies the use of racial categories as scientific and defines racism based on intention" (l. 299-301).*

Furthermore, reviewer #1 also suggested rephrasing the definition of 'pragmatism' to reflect that this type essentially includes the justification of diagnoses based on racial categorizations such as 'morbus mediterraneus'.

➔ *Here, too, we have rephrased the definition of this type accordingly. The respective definition now explains that the 'pragmatic' way of dealing with the topic of racism "orientates action towards tacit rules of clinical practice, justifies the use of racialised categories as practical and defines racism as an interpersonal problem." (l. 380-382).*

With regard to 'relativism', reviewer #1 found the definition that we provided rather unclear and demanded that we offer a more consistent definition. Also, the reviewer criticized that it was not clear why we called this type 'relativism', at all, and what the claim of not having enough knowledge about racism had to do with relativism.

➔ *We agree that this type is the most difficult to encapsulate in one short concise definition since it involves quite different aspects. Nevertheless, we rephrased the description given in order to make clear that this way of dealing with racism in discussions "stands for a lack of clear orientation of action and emphasizing uncertainty and subjective assessment when it comes to racialised categorisations as well as racism." (l.475-477) Furthermore, we agree with reviewer #1 that the label 'relativism' was a bit misleading for this type. Therefore, we have changed the label to 'subjectivist', reflecting that the respective way of dealing with the topic of racism involves the claim that racism is a matter of "subjective impressions and opinions" (l. 479).*

Reviewer #1 asked that we elaborate the discussion section in order to address participants' understanding of the relation between 'race' and racism, also in relation to other research in healthcare in this area.

➔ *We added elaborated discussions about how the different types of dealing with the topic of racism understand the relation of 'race' and racism, c.f. l. 775-784 for the 'scientistic' type A, l.*

785-792 for the 'pracmatic' type B, l. 800-804 for the 'subjectivist' type C, l. 817-822 for the 'interculturalis' type D, l. 830-833 for the 'critical' type D.

<u>Detailed comments</u>

1. **Reviewer #1 asked us to explain how the references provided l. 46 (now l. 49-52 in the unmarked copy) support the claim that discussion about racism decrease racist beliefs and discrimination.**

➔ *Evidence from medical education research shows that explicitly addressing racism and having discussions on this topic is an important step to tackle racism. We changed the sentence to "Talking about racism is an important precondition to induce reflection, decrease racist beliefs and discrimination, and expands critical action". We chose this more reluctant formulation since discussions about racism do not automatically lead to a decrease in racial bias and beliefs but can be an important precondition and an element of teaching activities about racism and antiracism in medicine and health care (this is what the cited literature conveys). To stress this, we added more English references, e.g. on simulation-based antiracist training in medical education that shows the importance of feedback and discussions on this topic or on the concept of white fragility that impedes discussions about racism (l. 50-52, endnotes 33-38).*

2. **Reviewer #1 asked that we provide the year of publication after Novak et al.**

➔ *We inserted the year of publication after Novak et al. (l. 56) and after all other in-text citations of this kind (e.g., l. 58, 62, 890)*

3. **Reviewer #1 pointed out that we misspelt the word 'Germany'.**

➔ *We changed the word accordingly (l. 66)*

4. **Reviewer #1 asked us to spell out 22 at the beginning of the sentence.**

➔ *We now spell out the number (l. 138).*

5. **Reviewer #1 advised that we include a review of research on racial bias under the section "Racism and anti-racism in medicine and healthcare"**

➔ *We included a longer review of research on racial bias in the respective section of the manuscript, covering major research strands and results in this field as well as different levels of medicine and healthcare, including individual bias as well as bias in knowledge bases and research (l. 110-136).*

6. **Reviewer #1 asked us to unpack the sentence l. 103 about the necessity to discuss racism in the context of habitus and suggested to introduce these terms earlier.**

➔ *We deleted the sentence and now instead introduce the relevant terms (professional socialisation, professional habitus) earlier (l. 42)*

7. **Reviewer #1 remarked that the analysis of the German CanMEDS framework should not be included in the introduction and could be the basis for another article.**

➔ *We deleted the part about antiracism and critical approaches to racism in the CanMed roles. Instead, we provided a definition of Anti-racism right in the beginning of the article (l. 35-38) and only kept the information on the competencies formulated in the NKLM (l. 151-156).*

8. **Reviewer #1 demanded that we revise the structure of table 1.**

➔ *We re-organized table 1 (l. 191). We used two PLOSone-articles related to the field of medical education as inspiration to revise the organization of the numbers in table 1 (Gottschalk et al. 2023; Tey et al. 2012). We added thematic headers for numbers concerning the same topic (gender, age, academic year) and added the type of numbers provided (n, range, mean). Moreover, by providing the classifying keywords from the participants' answers to the open-ended question about "experience of discrimination" in the pre-questionnaire, we made more transparent how we counted the participants' "experiences of discrimination".*

9. **Reviewer #1 asked whether minoritized and majoritized people were included in the same groups and how this influenced our methodological approach and the group discussions. The reviewer also asked us to explain why we did not conduct individual interviews with minoritized groups.**

➔ *Thank you for pointing out this crucial methodological aspect of our study. It is correct that people with and without personal experiences of racism were included in the same groups. We revised our methods section to explain why we opted for this research design instead of individual interviews with minoritised people: We were interested in observing how discussions in such diverse groups would develop. Discussions about racism do not only occur in specifically prepared teaching environments but often in clinical or educational context where this topic is not the main focus and group composition may be heterogenous (l. 168-173). Furthermore, in the discussion section, we elaborate our discussion of the influence of the diverse group composition on the discussions and our interpretation of the results. In particular, we explain how critical ways of dealing with the topic of racism sometimes remained without great validation and resonance in the group discussions. It is not that students explicitly discouraged other students to display a critical way to deal with the topic, instead, it was more implicitly through how statements related to other statements instead of taking up what was brought to the fore by students who employed a critical perspective on the topic (discussion: l. 868-871, results: 725-734)*

10. **Reviewer #1 pointed out that in line 324 (first submitted copy) we mention the Holocaust but no other genocides committed by Germans such as in Namibia.**

➔ *In this part of our result section (type A), we mention the Holocaust as part of the implicit and collectively shared knowledge that medical students refer to when they discuss the use of race categories in medicine in a 'scientific' way (l. 356-375). It is true that Germans participated in more racist crimes and genocides than the Holocaust, e.g. the Herero and Nama genocide in Namibia or the Romani Holocaust ('Porajmos') but the students only referred to the Holocaust in this sequence of the group discussions. Because of this, we decided to refrain from adding the genocide in Namibia as an external information to this section of the text.*

11. **Reviewer #1 wanted to know whether terms like 'Morbus mediterraneus' are actually used in medical curricula or rather are race talk used in lectures etc.**

- ➔ *We clarify that this is a colloquialism used in the everyday vernacular of clinical practice rather than in medical curricula (l. 407-409).*

12. **Reviewer #1 asked us to clarify how the descriptions of interculturalism provided in l. 521-8 as well as in l. 556-7 differ from the description of the previous types.**

- ➔ *We now explain that an interculturalist way of dealing with the topic of racism is based on the concern to overcome prejudices and to foster cultural sensitivity while a pragmatic way tends to accept cultural stereotypes as rough but handy tools for medical practice (l. 577-584).*

13. **Reviewer #1 asked us to include research on racial bias in healthcare in the discussion section and point out how our results contribute to this body of work.**

- ➔ *We now discuss our findings in the context of research on racial bias in medicine and healthcare. In doing so, we emphasize that our typology shows how individuals' specific ways of dealing with the topic of racism are the result of complex connections and negotiations of explicit and implicit orientations. This allows us to draw connections to other studies on bias and to point out how a habitus-based approach might help to provide additional an understanding of the connections between implicit biases as cognitive representations of acquired dispositions and their roots in social structures (l. 849-866).*

Academic Editor

The academic editor called upon us to tighten and improve the conceptual definitions, suggesting to stick to a more selective subset of concepts and provide more precise definitions of the concepts that are essential to our argument.

- ➔ *As already mentioned in our response to reviewer #1, we found this recommendation very helpful and made three major revisions in order to clarify and improve the manuscript's conceptual framework: We first reduced the number of concepts to the core conceptual framework, eliminating concepts that were not essential for our analysis, especially "pointed labels" (marked-up version: l. 380) or "heuristic tools" (marked up version: l. 568, 722, 981, 1077). Second, we now provide short working definitions of all the main concepts in the introduction: antiracism (lines 36-38), racialisation (l. 93-98), professional socialisation (l. 43-45), and habitus (line 46). Thirdly, we elaborate a bit further on these concepts and their interconnections in the methods section (l. 240-250), also providing a concise overview of our conceptual framework for analysis in the form of a table (l. 151).*

Furthermore, the academic editor asked us to strengthen the correspondence between the analytical claims we make and the empirical evidence we provide. The editor also recommended that we offer a more detailed discussion of how our insights are connected to broader scholarship on race outside the medical field.

- ➔ *In order to enhance the correspondence between analytical claims and empirical material, we reformulated the analytical descriptions of each type so that they encompass the main defining aspects that are unfolded in the concrete interpretation of the empirical material. In doing so, we*

could rely on very helpful hints and suggestions offered by reviewer #1. Thus, the definition of a 'scientistic' way of dealing with the topic of racism now includes that it "orientates action towards the idea of medicine as an objective science, justifies the use of racial categories as scientific and defines racism based on intention" (l. 299-301). The definition of a 'pragmatic' way now reflects that this way "orientates action towards tacit rules of clinical practice, justifies the use of racialised categories as practical and defines racism as an interpersonal problem." (l. 380-382). We also made clear that the subjectivist (formerly 'relativism') way of dealing with racism in discussions "lacks a clear orientation of action for dealing with the topic of racism and displays instead uncertainty and subjectivism in understanding racialised categorisations as well as racism." (l. 475-477) With regard to the discussion, we now highlight how our habitus-based research connects to broader research on racial bias and might help to provide an enhanced understanding of the connections between implicit biases as cognitive representations of acquired dispositions and their roots in social structures (l. 849-866).

Third, the academic editor asked us to enhance the validation and reflexivity statement in our manuscript to reflect our own subject positions as well as our relationship with the topic, participants and overall findings. With regard to validation, the editor kindly recommended pertinent work by Yardley.

➔ *We would like to thank the editor very much for this important advice. It gave us the opportunity to reflect our relation to the study and its participants in a much more profound and nuanced way. The manuscript now includes a considerably elaborated section on reflexivity, limitations and validation that touches upon the role of the white positionality of the research team, our relation as teachers and researchers to medical students as our research participants, as well as on the validation of our qualitative research with regard to Yardley's four dimensions of context sensitivity, commitment and rigor, transparency and coherence, and impact and importance (l. 899-968).*
