# Supplementary material for: How do medical students deal with the topic of racism? A qualitative analysis of group discussions in Germany
Source: PLoS One. 2024 Nov 19;19(11):e0313614. doi: 10.1371/journal.pone.0313614 (PMC11575774; doi:10.1371/journal.pone.0313614)
Supplement: Attachment — Submitted filename: Response to Reviewers_R2.pdf [file pone.0313614.s003.pdf]

Dear Dr. Emma Campbell and Dr. Joshua Uyheng,

Thank you for sharing the review of our manuscript “How do medical students deal with the topic of racism? A qualitative analysis of group discussions in Germany” (PONE-D-23-43307) and for giving us the opportunity to tackle remaining aspects that needed revision. We hereby submit the revised version together with a point-by-point response. We hope the piece now meets the requirements of PLOSone and look forward to hearing from you.

Kind regards,

The authors

Reviewer #1

Reviewer#1 stated that all comments have been addressed and made no further comments.

We would like to thank again Reviewer 1 for the detailed and very instructive review in round one. We are glad to hear that we addressed all points raised.

Academic Editor

The academic editor asked us to ensure that the title is consistent and pointed to the fact that there are different versions in the cover letter and the submitted manuscript. The editor sees the version that reads "How do medical students deal..." as preferable.

Thank you for pointing out this inconsistency. We had changed the manuscript title in the first round of revisions but still used the original title in the response letter. We now consistently use the new title “How do medical students deal with the topic of racism? A qualitative analysis of group discussions in Germany” that is found in the revised manuscript and this response to the reviewers. Accordingly, we also edited in the supporting information S1_Text (discussion guideline) that is intended to be published with the article and uploaded a revised version.

The academic editor also suggested that the italicization of white (when referring specifically to the authors' racial identification) be omitted.

Thank you for this suggestion. We followed the suggestion and deleted the italicization of “white” throughout the document.

Journal requirements

The journal asked us to review our reference list to ensure that it is complete and correct and to mention any changes to the reference list in the rebuttal letter. Especially the citation of retracted articles should be indicated as such in the References list and also include a citation and full reference for the retraction notice.

We checked again our reference list to ensure that it is complete and correct. We do not cite a retracted article. The article cited as reference no. 135 has been corrected but not retracted. We made the following changes to the reference list:

- *Reference no. 2: Added “th” to “7th ed.".*
- *Reference no. 6: Deleted “Policy & Procedure Manual” in the title., deleted “Canada” and edited the date published and cited.*
- *Reference no. 14: Edited Date according to format style.*
- *Reference no. 15: Added interpunctuation.*
- *Reference no. 25: Added “p” to “pp”*
- *Reference no. 26: Added “p” to “pp”*
- *Reference no. 28: Added "p” to “pp”*
- *Reference no. 30: Deleted unnecessary “1.”, added “p” to “pp”*
- *Reference no 32: Added DOI*
- *Reference no. 33: Deleted “Wiesbaden” in publisher’s name, added “p” to “pp”*
- *Reference no. 37: Edited capitalisation of the title*
- *Reference no. 39: Added “p” to “pp”*
- *Reference no. 40: Added “p” to “pp”*
- *Reference no. 45: Corrected page numbers and added “p” to “pp. 205-215”.*
- *Reference no. 51: Added “p” to “pp”*
- *Reference no. 52: Adeed “p” to “pp”*
- *Reference no. 66: Deleted PubMed ID*
- *Reference no. 67: Deleted PubMed ID*
- *Reference no. 68: Corrected typo in title, deleted “A” in publisher’s name.*
- *Reference no. 70: Corrected typo in “USA”*
- *Reference no. 76: Deleted “[1952]” as first published date according to the citation guidelines.*
- *Reference no. 78: Added DOI*
- *Reference no. 82: Added DOI*
- *Reference no. 93: Deleted PubMed ID*
- *Reference no. 97: Deleted Epub date.*
- *Reference no. 116: Edited capitalisation of the edited book’s title, deleted unnecessary parts of the edited book’s subject, added “p” to “pp”*
- *Reference no. 120: Added “p” to “pp”*
- *Reference no. 123: Added “p” to “pp”*
- *Reference no. 133: Added editor and edited capitalisation of the edited book’s title. Added place published and publisher. Added “p” to “pp”.*
- *Reference no. 137: Changed the journal’s title to the abbreviated form (ISO 4)*
- *Reference no. 137: Added “p” to “pp”*
- *Reference no. 138: Added “p” to “pp”*
- *Reference no. 140: Edited incorrect information about the journal’s title and changed the correct journal’s title to the abbreviated form (ISO 4): “Am Psychol”*
