# Supplementary material for: How do medical students deal with the topic of racism? A qualitative analysis of group discussions in Germany
Source: PLoS One. 2024 Nov 19;19(11):e0313614. doi: 10.1371/journal.pone.0313614 (PMC11575774; doi:10.1371/journal.pone.0313614)
Supplement: Attachment — Submitted filename: Response to Reviewers_R3.pdf [file pone.0313614.s005.pdf]

Response to Reviewers PONE-D-23-43307

Dear Dr. Rahul Sambaraju,

Thank you for sharing the reviews of our manuscript "How do medical students deal with the topic of racism? A qualitative analysis of group discussions in Germany" (PONE-D-23-43307) and for giving us the opportunity to tackle remaining aspects that needed revision. We hereby submit the revised version together with a point-by-point response. We hope the piece now meets the requirements of PLOS ONE and look forward to hearing from you.

Kind regards,
The authors

Reviewer #1 (Sarah Hamed)

Reviewer#1 confirmed that all comments had been addressed in the previous round of revisions and underlined that the article will help readers understand the production of racialisation in healthcare.

We would like to thank Reviewer #1 again for the detailed and very instructive review in round one. We are glad she evaluated our article positively.

Reviewer #2

Reviewer #2 summarized the articles' content, gave overall positive feedback, and suggested minor revisions to address five aspects.

We thank Reviewer #2 for the encouraging feedback and helpful comments that point to important aspects. Addressing them helped us improve and clarify the article's argument.

1. **Regarding the background section, Reviewer #2 recommended clarifying the argumentative transition from Fanon's perspective on "implicit knowledge" on the one hand and the more cognitive take on physicians' racist behaviour with the concept of "implicit bias". Moreover, Reviewer #2 proposed returning to the relevance of implicit bias research compared to systemic explanations in the discussion.**

Thank you for the close reading and for identifying this argumentative weakness in the article's background section. We tackled this inconsistency by linking Fanon's analysis of "implicit knowledge" more explicitly to the concept of habitus that connects the individual and collective/structural aspects of racism (105-113). Moreover, to distinguish this perspective from the one of implicit bias research, we now begin the paragraph about implicit bias with the explanation that research on implicit bias "takes a closer look at the cognitive processes […]"

(114-115) involved in racism. Furthermore, we added another sentence in the discussion section highlighting the importance of analysing structural aspects for understanding the persistence of racism in medicine and health care (861-863).

2. **Regarding the article's methods section, Reviewer #2 suggested mentioning who translated the quotes from the material.**

Thank you for this suggestion! We added the information in line 234: "the quotes from the material were translated into English by the authors."

3. **Reviewer #2 shared the impression that calling the five identified ways of dealing with the topic of racism a 'typology' evokes the impression of them being "fixed" with no possibility to change or adapt. Reviewer #2 recommends choosing a different wording. They also suggest calling the first identified way of dealing with the topic of racism "scientist" rather than "scientistic."**

We have revised the whole text to avoid terminology suggesting a systematic typology with fixed types. Instead, we now speak of typical ways, approaches, or perspectives in dealing with racism. However, after careful consideration, we decided to stick to the term "scientistic" for three reasons: First, as Reviewer #2 noted, we use adjectives for all five ways of dealing with the topic of racism and would like to keep this nomenclature consistent. Secondly, we think this use of adjectives actually helps to signal that we analyse typical "ways" (modes of talking and acting) rather than fixed types of people. Thirdly, we are concerned that the term "scientist" might be misleading since it might suggest a scientific approach to racism. We think "scientistic" (derived from the position of scientism) makes clearer that we talk about a professional habitus that refers to (a particular understanding of) science to justify positions about racism and lend them authority.

4. **Regarding the discussion, Reviewer #2 suggested changing how we report the findings from another study about the hypersexualization of black women. Instead of writing about the experience of black women to be hypersexualised, Reviewer #2 proposes to change the language to make clear that it is not only an experience of black women but something that in fact happens.**

We changed the sentence according to Reviewer #2's suggestion. It now reads: "Black women are hypersexualized and more likely to be checked for STIs" (851).

5. **Regarding the discussion, Reviewer #2 also suggested discussing the need for more interdisciplinary collaboration and research with special regard to fostering the understanding of racism as a system by including, e.g., sociological perspectives into medical education.**

Thank you for this important point! We came to the same conclusion and, therefore, added an explicit statement about the need to include more institutionalized interdisciplinarity in medical education (1008-1011).
