# Supplementary material for: How do medical students deal with the topic of racism? A qualitative analysis of group discussions in Germany
Source: PLoS One. 2024 Nov 19;19(11):e0313614. doi: 10.1371/journal.pone.0313614 (PMC11575774; doi:10.1371/journal.pone.0313614)
Supplement: Attachment — Submitted filename: Article review.docx [file pone.0313614.s004.docx]

**Review on “How do medical students deal with the topic of racism?”**

Dear colleagues, I read your study with great interest. Below is the summary of the article with my impressions. I then give specific suggestions that I would recommend you address in your revision.

This article analyzes German-speaking medical students speaking about the topic of racism in online group conversations. The authors provide a good introduction and literature review that introduces how racism, race, and bias is thought about in this article. The qualitative study using virtual focus group interview data is relevant to the research questions as they give both a thematic overview of the ideas that medical students have related to questions of racism, and also show how medical students explain their understanding of racism and race. The results are presented in a clear manner and use relevant quotes, and the tables support the results well. The discussion and conclusion of the paper has some useful takeaways.

Based on this positive assessment, I have some comments that I think are important for the authors to take into account, and I have some suggestions that you can consider:

1. Literature review: The discussion of racism as an ideology and system is well argued. Paying attention to the implicit forms of racist meaning making is an important point. However, I’d make the transition from Fanon’s point to racial bias more clear (110 to 111). I think that these two interpretations are slightly different, and that moving from Fanon’s explanation that racism structures “implicit knowledge” (line 108) is not the same thing as implicit bias which connects more to a cognitive argument of explaining racism. Please make this transition more clear, and articulate why you see both of these explanations as necessary. Also, you could return to this question of implicit bias in the conclusion - and discuss if this still hold its relevance or if other explanations, like systemic ones might be more relevant.
2. Methodology: Write who translated the quotes from your material.
3. Findings: The findings are clear and the quotes relevant. However, I’ve got some questions/concerns regarding the types. I don’t think that a typology of how medical students deal with racism is a helpful description of what’s happening. Rather than types, as you say yourself, these are 5 typical ways that German-speaking medical students deal with the topic of racism; and each person could potentially adapt each way. I suggest that you don’t use the word types to describe it but something else, for example explanations. The term type evokes the idea of typology, which can be associated with fixity, an inability to change. I think an important point that you’re making by using the approach of habitus is that we are socialized into certain behaviors and thoughts, and that we can also change. I think that this should be reflected in the naming of the 5 typical ways. One other word I don’t find so fitting is “scientistic.” I understand that you want to use adjectives to describe each typical way, but I think saying “scientist” would be sufficient.
4. Discussion: This is a good discussion in which you discuss the main takeaways. I wonder if the language could be changed slightly in lines 845-847. You write that “Black women experience to be hypersexualised…” By writing experience to be, I get the impression that this could be debatable. I suggest that you instead write “Black women are hypersexualized” to demonstrate that this in fact happens, and it is not just from their experiences. Finally, it might add to your article to discuss specifically the need for more interdisciplinary work within medical education. Specifically, the lack of critical literacy in terms of understanding racism as a system demonstrates the need for more education outside of the “hard” sciences.

I hope that these requests and suggestions are helpful for the authors to improve the quality of this study.
